# 3D Bioprinting of Model Tissues That Mimic the Tumor Microenvironment

**DOI:** 10.3390/mi12050535

**Published:** 2021-05-09

**Authors:** Florina Bojin, Andreea Robu, Maria Iulia Bejenariu, Valentin Ordodi, Emilian Olteanu, Ada Cean, Roxana Popescu, Monica Neagu, Oana Gavriliuc, Adrian Neagu, Stelian Arjoca, Virgil Păunescu

**Affiliations:** 1 Department of Functional Sciences, Victor Babes University of Medicine and Pharmacy Timisoara, 300041 Timisoara, Romania; florinabojin@umft.ro (F.B.); olteanu.gheorghe@umft.ro (E.O.); popescu.roxana@umft.ro (R.P.); neagu.monica@umft.ro (M.N.); oana_gav@yahoo.com (O.G.); neagu@umft.ro (A.N.); vpaunescu@umft.ro (V.P.); 2 OncoGen Institute, 300723 Timisoara, Romania; valentin.ordodi@upt.ro (V.O.); teleaada@gmail.com (A.C.); 3 Department of Automation and Applied Informatics, “Politehnica” University of Timisoara, 300223 Timisoara, Romania; andreea.robu@aut.upt.ro; 4 Faculty of Mechanical Engineering, “Politehnica” University of Timisoara, 300222 Timisoara, Romania; bmaria.iulia@gmail.com; 5 Department of Microscopic Morphology-Morphopathology, ANAPATMOL Research Center, Victor Babes University of Medicine and Pharmacy Timisoara, 300041 Timisoara, Romania; 6 Center for Modeling Biological Systems and Data Analysis, Victor Babes University of Medicine and Pharmacy Timisoara, 300041 Timisoara, Romania; 7 Department of Physics and Astronomy, University of Missouri, Columbia, MO 65211, USA

**Keywords:** breast cancer, tumor-associated fibroblasts, peripheral blood mononuclear cells, extrusion bioprinting

## Abstract

The tumor microenvironment (TME) influences cancer progression. Therefore, engineered TME models are being developed for fundamental research and anti-cancer drug screening. This paper reports the biofabrication of 3D-printed avascular structures that recapitulate several features of the TME. The tumor is represented by a hydrogel droplet uniformly loaded with breast cancer cells (10^6^ cells/mL); it is embedded in the same type of hydrogel containing primary cells—tumor-associated fibroblasts isolated from the peritumoral environment and peripheral blood mononuclear cells. Hoechst staining of cryosectioned tissue constructs demonstrated that cells remodeled the hydrogel and remained viable for weeks. Histological sections revealed heterotypic aggregates of malignant and peritumoral cells; moreover, the constituent cells proliferated in vitro. To investigate the interactions responsible for the experimentally observed cellular rearrangements, we built lattice models of the bioprinted constructs and simulated their evolution using Metropolis Monte Carlo methods. Although unable to replicate the complexity of the TME, the approach presented here enables the self-assembly and co-culture of several cell types of the TME. Further studies will evaluate whether the bioprinted constructs can evolve in vivo in animal models. If they become connected to the host vasculature, they may turn into a fully organized TME.

## 1. Introduction

Cancer, as a cause of death, is only surpassed by cardiovascular diseases [1]. According to the World Health Organization, in 2020, there were 19.3 million cases worldwide, and about 10 million people died of cancer. Between 2005 and 2015, the number of oncology patients increased by 33%, mainly due to population aging and population growth. Nevertheless, cancer mortality decreased in the same period in 140 of 195 countries or territories monitored in the Global Burden of Disease 2015 Study, demonstrating that progress is being made in cancer treatment and prevention [1].

Given the complexity and heterogeneity of cancer, various therapeutic targets are being investigated, including components of the tumor milieu. The tumor microenvironment (TME) consists of several types of cells (tumor-associated fibroblasts, immune cells, mesenchymal stem cells, adipocytes, and vascular cells) embedded in extracellular matrix (ECM) soaked by interstitial fluid rich in soluble factors secreted by cells [2].

A compelling body of evidence indicates that tumor progression depends on the interaction between the tumor and its microenvironment [3]. It is known that the effectiveness of anti-cancer therapies is modulated by changes in the TME [2,4]. Therefore, extensive research efforts have been devoted to investigating the spatial organization of the native TME [5] and to build in vitro models of the TME using three-dimensional (3D) bioprinting [6] and lab-on-a-chip techniques [7].

Conventional cancer models, such as 2D cultures of cancer cells and stromal cells, cannot mimic the TME, especially in what concerns oxygen and nutrient availability, thereby being limited as biomimetic models for anti-cancer drug testing or fundamental research in cancer biology [8]. The lack of tumor-stroma interaction is also a weak point in patient-derived tumor organoid models otherwise highly valued for their ability to replicate intra- and intertumor heterogeneity in high-throughput cultures of 3D constructs [9].

Bioprinting enables the precise positioning of cells and biomaterials in a computer-controlled, layer-by-layer procedure that preserves cell viability [10]. Therefore, it is considered a promising avenue towards building tissue constructs that recapitulate the compositional and geometric complexity of the TME [11,12,13,14,15,16,17,18,19,20]. Although none of the model tissues constructed so far have been able to replicate all the features of a TME, most of them provided valuable insights into cancer biology. For example, in a rectangular grid of hydrogel threads uniformly loaded with HeLa cells from a cervical adenocarcinoma cell line, multicellular spheroids formed in less than fivedays of culture, which proved to be more resistant to paclitaxel (an antineoplastic chemotherapy drug) than HeLa cells in 2D culture [20]. In addition, a bioprinted cancer-on-chip model recapitulated the biochemical and biophysical features of glioblastoma and reproduced the clinically observed, patient-specific variability of the response to concurrent administration of chemotherapy and radiotherapy [18].

The present work aimed to develop 3D bioprinting methods for building in vitro models of the TME made of cells uniformly distributed in an artificial hydrogel matrix.Our working hypothesis was that the tumor couldbe represented by a cancer cell-laden hydrogel droplet, whereas its microenvironment can be modeled by rings of a hydrogel loaded with peritumoral cells. To our knowledge, this is the first report of a nanocellulose-alginate hydrogel being used for the bioprinting of breast cancer models. In this study, we included tumor-associated fibroblasts (TAFs) and, for the first time, peripheral blood mononuclear cells (PBMCs) in the TME model. The design of this study is represented schematically in the Supplementary Material (Figure S1).

TAFs are the most abundant stromal cell type in various solid tumors (e.g., breast, pancreatic, and colorectal) [21,22]. They synthesize most of the tumor’s ECM and modulate tumor progression by secreting growth factors, cytokines, chemokines, and matrix-degrading enzymes [23]. In mouse models of pancreatic and lung carcinoma, it has been established that TAFs block intratumoral infiltration of lymphocytes by highly expressing CXC-chemokine ligand 12 [2]. Hence, TAFs also play an immunosuppressive role. There is solid evidence that TAFs originate from bone marrow-derived MSCs [24] and present immunophenotypic similarities with them [25]. Recent works, however, suggest that TAFs emerge from cancer stem cells, a subpopulation of tumor cells capable of self-renewal and differentiation [26,27].

PBMCs are a heterogeneous population of immune cells. The co-culture of PBMCs and epithelial tumor organoids allowed for the expansion of tumor-reactive T-cells [28]. We chose to include PBMCs in the TME tissue constructs because several studies have demonstrated that the clinical progression of cancer depends on the level of immune cell infiltration of tumors. A strong lymphocytic infiltration was found to be associated with longer disease-free survival (after surgery) and/or overall survival in many types of cancer, including melanoma, head and neck, breast, prostate, colon, bladder, and lung cancer [29]. In lung cancer patients, the gene expressionprofile of PBMCs measured before tumor resection was found to be an independent predictor of patient survival: higher expression of genes associated with protein synthesis was an indicator of better survival, whereas higher expression of genes associated with cell cycle was linked to worse survival [30].

Besides the characterization of the bioprinted tissue constructs, this work also presents computer simulations of post-printing structure formation. Such simulations are necessary because cells take advantage of their motility to form clusters of optimal stability and function [31]. Hence, 3D bioprinting merely provides a framework for the directed self-assembly of the cells delivered by the printer.

A notable limitation of the TME model proposed here is the lack of endothelial cells in the peritumoral medium. The motivation of this option is twofold: (i) it allows for the optimization of the co-culture conditions for cancer cells, TAFs and PBMCs, and (ii) it differentiates this study from previous investigations [11], thereby providing complementary information. From the immunological point of view, the TME model avoids the possible interactions between tumor cells and immune cells (PBMCs) based on non-self recognition, leading to a cytotoxic and destructive mechanism. This was possible due to the selection of TME-comprising cells to be major histocompatibility complex (MHC)-compatible with the tumor cells. This makes the proposed model more reliable for replicating in vivo tumor development.

## 2. Materials and Methods

### 2.1. Preparation of Malignant Cells and Peritumoral Cells

#### 2.1.1. Culturing SKBR3 Cells

Tumor cells from SK-BR-3 cell line (ATCC^®^ HTB-30™, Lomianki, Poland) were cultured in McCoy’s 5a medium modified (Gibco BRL, Invitrogen, Carlsbad, CA, USA) supplemented with 10% fetal calf serum (FCS, PromoCell, Heidelberg, Germany) and 1% penicillin/streptomycin antibiotic solution (Pen/Strep, 10,000 IU/mL; PromoCell) using adherent culture flasks and incubated at 37 °C in 5% CO_2_ atmosphere. When reaching 90% confluence, the cells were detached from the plastic surface with 0.25% (*w/v*) trypsin EDTA solution (Sigma-Aldrich, St. Louis, MO, USA) and further expanded at a subcultivationratio of 1:2, as recommended by the provider’s protocol.

#### 2.1.2. Harvesting and Culturing Tumor-Associated Fibroblasts (TAFs)

Human TAFs were isolated using the collagenase type IV-S prepared from *Clostridium histolyticum* (Sigma-Aldrich), as previously described by Paunescu et al. [25]. In brief, surgical pieces of approximately 5 cm^2^ were obtained from breast cancer female patients diagnosed in different carcinoma stages. Tissue-isolated cells were washed several times with phosphate-buffered saline (PBS) solution, were successively passed through 70/40 µm strainer filters and were cultivated in adherent plastic culture plates. The culture medium contained α-minimum essential medium (α-MEM; Gibco), 10% fetal calf serum (FCS; PromoCell) and 1% antibiotics solution (penn/strep, 10,000 IU/mL;PromoCell), and the tumor-associated fibroblasts were placed in cell culture incubator (37 °C and 5% CO_2_).

#### 2.1.3. Harvesting and Culturing PBMCs

PBMCs were obtained from 10 mL venous peripheral blood by centrifugation in a density gradient. The blood sample was harvested from the breast cancer patients before the surgical intervention in EDTA collection tubes. For PBMCs isolation, we used 10 mL Ficoll-Paque PLUS (Sigma-Aldrich), which was placed on the bottom of a 50 mL Falcon tube, while on top, we slowly pipetted the peripheral blood diluted with PBS (Gibco) at a ratio of 1:1. We collected the mononuclear cells ring after centrifugation at 500× *g* and deceleration 0 for 25 min. After washing the PBMCs twice with PBS (Gibco), the cells were cryopreserved in liquid nitrogen (−196 °C) in a medium containing FCS (PromoCell) and 10% dimethyl sulfoxide (DMSO; Sigma-Aldrich), at a concentration of 10^6^ cells/mL, for further use.

The blood samples were submitted for HLA-typing in an independent study, and we selected only the PBMCs and TAF from patients with HLA type A*11 to be compatible with the SK-BR-3 tumor cell line.

All tissue and biological samples were obtained after signing the informed consent elaborated under a protocol approved by the Ethical Commission of the County Emergency Hospital “Pius Brinzeu” Timisoara, according to the World Medical Association Declaration of Helsinki.

### 2.2. Three-Dimensional Bioprinting of Model Tissues

In this study, we employed an INKREDIBLE bioprinter (CELLINK, Gothenburg, Sweden) to deliver cell-laden hydrogel strands in a computer-controlled arrangement at 10 µm in-plane resolution, and 100 µm layer resolution. This bioprinter is equipped with two pneumatic print heads, whose extrusion rate depends on the pressure applied to the piston, the geometry of the extrusion nozzle and the rheological properties of the bioink. An external compressor, connected to a different outlet than the bioprinter, provides pressurized air at a pressure of up to 250 kPa. The pressure applied to each print head can be set at a fraction of this pressure via two dials. Using blunt needles of 0.6 mm internal diameter as print nozzles, typical print-head pressures employed in our experiments ranged between 20 kPa and 100 kPa.

#### 2.2.1. Preparation of Cell-Laden Hydrogels

For the preparation of cell-laden hydrogels, the three cellular types were processed individually according to their specific requirements. The adherent cells (TAFs and SK-BR-3 cells) were processed using the trypsinization method. Shortly, when reaching 90% confluence, the culture flasks were washed with warm PBS (Gibco), and 0.25% (*w/v*) trypsin EDTA solution (Sigma-Aldrich, St. Louis, Mo, USA) was added, 1 mL/1 cm^2^ of culture surface. After centrifugation (10 min, 500× *g*), the cells were resuspended in a culture medium and counted. The cryopreserved PBMCs were thawed, centrifuged (10 min, 500× *g*) and counted. Then, TAF and PBMCs were mixed in the same cellular suspension, as they were printed with the same printing head. The cellular mixture between TAF and PBMCs contained 1.5 × 10^6^ cells of each type, suspended in 300 µL of RPMI (Gibco), 10% FCS (PromoCell) and 1% antibiotics solution (penn/strep, 10,000 IU/mL;PromoCell). The SK-BR-3 cells were also resuspended in 300 µL of the same culture medium. Using the cell mixer kit (CELLINK), 3 mL of CELLINK Bioink (universal bioink, CELLINK) was used for uniformly mixing 300 µL of TAFs and PBMCs (3 × 10^6^ cells), following the provider’s protocol; other 3 mL of CELLINK were mixed with 300 µL of SK-BR-3 cells (3 × 10^6^ cells), following the same protocol. As a result of the mixing procedure, we obtained two printing cartridges of 3 mL containing cell-laden hydrogel: one with TAFs and PBMCs mixed in equal proportions and the other with SK-BR-3 cells. In both cartridges, the cellular density was 10^6^ cells/mL.

#### 2.2.2. Digital Modeling and Bioprinting

The Rhinoceros^®^software(Robert McNeeland Associates, Seattle, WA, USA) was used to build digital models of two tissue constructs. The first one, called hereafter the toroidal structure, consists of a spheroid tightly embraced by a donut-shaped hydrogel ring; the second, the so-called triple-layered construct, comprises the toroidal structure sandwiched between two hydrogel layers—a pair of concentric rings at the bottom and one ring at the top (see Supplementary Materials, Figure S2). The pyramid shape of the triple-layered construct is advantageous because it ensures the stability of the hydrogel rings stacked on top of each other; therefore, this geometry is more reproducible than a cylindrical or spherical one.

We did not rely on a slicer software to generate the G-code needed to control the bioprinter. Instead, we employed the digital model as a template for writing the G-code, thereby retaining full control of the path and movement speed of the print head.

During printer calibration, we ascertained that the first print head nozzle was 0.1 mm above the target surface. By printing straight lines at the feed rate used in the actual bioprinting process, we identified the air pressure applied to the piston of the print head for each type of hydrogel employed in our experiments. The pressure was adjusted until the printer delivered a uniform gel strand about 20% higher in diameter than the inner diameter of the extrusion nozzle.

**Figure 1:**
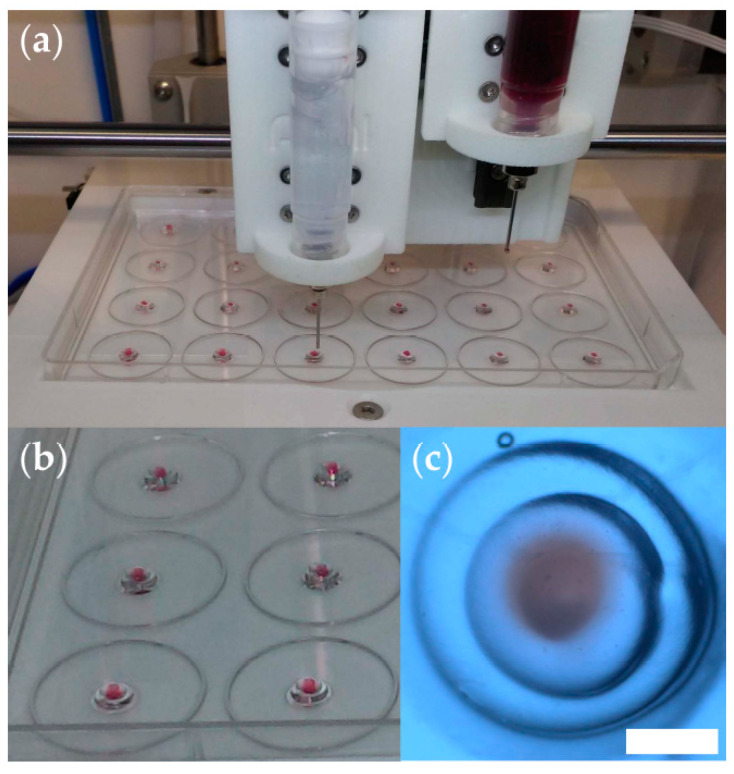
Optimization of the 3D bioprinting procedure. (**a**) Snapshot of a test print using the INKREDIBLE bioprinter to deliver hydrogel constructs onto the lid of a 24-well cell culture plate; here, we used ultrasound transmission gel to model the peritumoral medium, and the same gel stained with neutral red dye to model the tumor; (**b**) photograph of triple-layered structures obtained in test-prints. (**c**) Stereomicroscopy image (top view) of a representative triple-layered structure (scale bar = 1 mm).

To deliver a hydrogel ring, we employed the G1 command, substituting circular arcs with the corresponding chords (i.e., a circle was written as a succession of 12 straight lines), but the result was a smooth ring due to bioink viscosity (Figure 1a). The feed rate was set to 150 mm/minute during extrusion and 900 mm/minute during the repositioning of the print head from one construct to another. Before drawing a circle, a dwell time of 0.3 s was allocated, via the G4 command, to initiate hydrogel extrusion. We used Konix ultrasound transmission gel (Turkuaz, Istanbul, Turkey) in testprints meant to establish the settings of the bioprinter. The tumor was modeled by Konix gel stained with Neutral Red dye (Sigma-Aldrich, Hamburg, Germany). Supplementary Video S1 illustrates the entire printing process.

Figure 1 illustrates the reproducibility of the 3D printing process. As shown in Figure 1c, the tissue construct is about 3 mm in diameter, and the central spheroid (red) is about 0.8 mm in diameter. The actual bioprinting took place in the 24-well cell culture plate, and the tall walls of the wells hampered visualization (Figure S3).

### 2.3. In Vitro Culture of Model Tissues

The 3D bioprinted tissue constructs were placed individually in wells of a 24-well culture plate and grown for 14 days in a medium containing RPMI (Gibco), 10% FCS (PromoCell) and 2% antibiotics solution (penn/strep, 10,000 IU/mL;PromoCell). The medium selection reflected the constructs composition of three cell types that can be cultivated in RPMI, but we increased the amount of the antibiotic as the constructs suffered multiple processing steps.

### 2.4. Histology

TAF immunocytochemistry staining was performed as previously described [25] on adherent cells for confirming their phenotype, using the following monoclonal mouse anti-human primary antibodies from Dako company (Carpinteria, CA, USA): alpha-smooth muscle actin (α-SMA), CD105 (endoglin; clone SN6h), vimentin (clone VIM 3B4), and pan-cytokeratin (Pan-CK; AE1/AE3).

The bioprinted tissue constructs were sectioned and evaluated by two histological methods: Hoechst and hematoxylin–eosin (HE) staining of cryosections [32].

Cryosections were prepared from tissue constructs cultured for 14 days. The samples were covered in tissue freezing medium (Leica Biosystems, Wetzlar, Germany), snap-frozen in liquid nitrogen and sectioned at 5 µm thickness using a Leica CM 3050 S Cryostat (Leica) cryotomy instrument. The cryosections were treated with Hoechst 33342 (Invitrogen) nucleic acid stain, and a cover slide was added using Fluoromountaqueous mounting medium (Sigma-Aldrich). When the HE stain was used, the hematoxylin solution (Sigma-Aldrich) colored the nuclei of the cells for 2 min, and the eosin (Sigma-Aldrich) was placed to stain the cellular cytoplasm for 30 s, followed by distilled water washing procedures. The HE stained sections were mounted with gel mount aqueous mounting medium (Sigma-Aldrich). The sections stained with either Hoechst or HE were visualized using ZEISS Axio Observer microscope (Zeiss, Munich, Germany).

### 2.5. Computational Modeling of the Evolution of Model Tissues

To simulate the evolution of bioprinted model tissues, we incorporated a new algorithm in the *SIMMMC* application [33]. This algorithm is an extension of the “SIMMMC for bioprinting” module [34], which describes shape changes of live systems made of two types of cells in an environment that contains a cell culture medium, a hydrogel that can be remodeled by cells, as well as a biomaterial that cannot be penetrated or altered by cells. Besides the Metropolis Monte Carlo (MMC) algorithm [35,36], the extended module also includes events that account for cell proliferation.

Our computational model is built on a 3D cubic lattice. Each lattice site is occupied by a point particle that stands for a cell or a similar-sized volume element of one of the three possible embedding materials included in the model. Site occupancy is specified by a particle-type index, σ, as follows: σ=0 for medium, σ=1 for hydrogel, σ=2 for peritumoral cells, σ=3 for cancer cells, and σ=4 for the biomaterial. The latter, however, was not present in the experiments conducted in this study; therefore, the simulations only involved particles of type 0, 1, 2, and 3.

In the model system, interactions are described in terms of works of cohesion, $\varepsilon_{\sigma \sigma }$, and works of adhesion, $\varepsilon_{\sigma \sigma \prime }$ (a term used when $\sigma \neq \sigma^{\prime }$); these are defined as the mechanical work needed to break the bond between two adjacent particles whose types are specified by the pair of indices [37]. The total energy of adhesion can be written as [38]:

(1) $$E=\sum_{\begin{array}{l} \sigma ,\sigma^{\prime }=0\\ \sigma <\sigma^{\prime } \end{array}}^{3}\gamma_{\sigma \sigma^{\prime }}N_{\sigma \sigma \prime }-\frac{1}{2}n_{n}\sum_{\sigma =0}^{3}\varepsilon_{\sigma \sigma }N_{\sigma },$$

where $n_{n}$ = 26 is the number of up to second-nearest neighbors of a given lattice site—called hereafter the neighbors of the site; $N_{\sigma \sigma^{\prime }}$ is the number of heterotypic bonds between particles of type σ and $\sigma^{\prime }$; $N_{\sigma }$ is the number of particles of type σ, and $\gamma_{\sigma \sigma^{\prime }}=\left(\varepsilon_{\sigma \sigma }+\varepsilon_{\sigma^{\prime }\sigma^{\prime }}\right)/2\;\;-\;\varepsilon_{\sigma \sigma^{\prime }}$ is the interfacial tension of the surface of separation between the two media, of type σ and $\sigma^{\prime }$. In Equation (1), the first term is the total interfacial energy of adhesion, whereas the second term is the total energy of cohesion.

We simulated the evolution of the bioprinted TME models using the following computational algorithm:

1. Compute the total energy of adhesion E, given by Equation (1);
2. Commence the first Monte Carlo step (MCS) by identifying interfacial cells (i.e., particles of types 2 and 3 with neighbors of a different kind);
3. Take type 3 (cancer) cells, in a random order, and give them the chance to proliferate with a probability $p_{3}$ or to move (i.e., swap with a neighbor of a different type);
4. Pick type 2 (peritumoral) cells in a random sequence, and give them the chance to proliferate with a probability $p_{2}$ or to move (i.e., swap with a type 1 or a type 0 particle—note that allowing a type 2 particle to also swap with a type 3 particle would lead to a repeated rearrangement of the corresponding interface);
5. If the chosen event is proliferation, create a new cell of the same type by replacing a randomly picked neighbor of type 1 or 0;
6. If the event of choice is movement, compute the corresponding change in energy, ΔE, and accept the move if ΔE≤0; otherwise, accept it with a probability $P=\exp\left(-\Delta E/E_{T}\right)$;
7. Increment the number of MCS by one and start a new MCS.

In the expression of the trial move acceptance probability, P, (step 6 of the algorithm), $E_{T}$ is the biological analog of the energy of thermal fluctuations from statistical physics, equal to Boltzmann’s constant multiplied by the absolute temperature; this quantity is a surrogate measure of cell motility [39]. It is convenient to express works of adhesion and interfacial tensions in units of $E_{T}$.

## 3. Results

**Figure 2:**
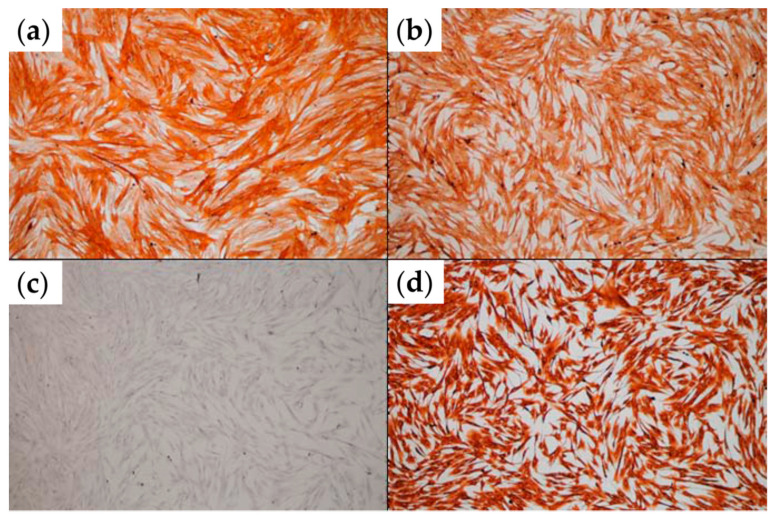
Immunocytochemical analysis of TAFs expanded in 2D culture. (**a**) α-SMA staining; (**b**) CD105 staining; (**c**) pan-CK staining; (**d**) vimentin staining (optical microscopy, ob. 10×).

Confluent monolayers of primary tumor-associated fibroblasts (TAFs) were analyzed via immunocytochemistry. Figure 2 demonstrates that, as expected, the TAFs employed in this study expressed alpha-smooth muscle actin (α-SMA), CD105 and vimentin, but no pan-cytokeratin (Pan-CK), characteristic markers for this cellular type [25].

### 3.1. Histological Evaluation of Bioprinted Tissue Constructs

**Figure 3:**
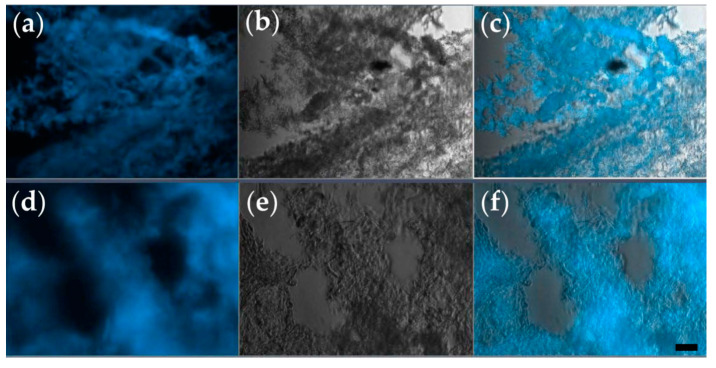
Representative cryosections of tissue constructs, prepared after 2 weeks of in vitro culture. (**a**–**c**) Section of the toroidal tissue construct; (**d**–**f**) section of the triple-layered tissue construct. Each section is visualized as a fluorescence microscopy image obtained via Hoechst staining (**a**,**d**), bright-field microscopy (**b**,**e**), and their overlay (**c**,**f**); (ob. 10×); scale bar = 10 µm.

The Hoechst nucleic acid stain is a cell-permeant stain that generates blue fluorescence when bound to double-stranded DNA. Therefore, it serves to visualize the arrangement of cells within a tissue section (Figure 3a,d).

As shown in Figure 3, after 14 days of in vitro culture, both types of tissue constructs contain regions densely populated by cells, and these regions alternate with cell-free domains. Such a distribution of cells is consistent with the hypothesis that the cells grow, proliferate, and form aggregates around protein filaments present in the CELLINK hydrogel. Although initially, both types of bioprinted constructs contained 10^6^ cells/mL, the cell density after 2 weeks was higher in cell clusters formed in triple-layered constructs (Figure 3e,f) than in donut-shaped, toroidal constructs (Figure 3b,c). Furthermore, triple-layered structures were superior to toroidal ones also in the consistency of cellular arrangements (compare panels c and f of Figure 3).

**Figure 4:**
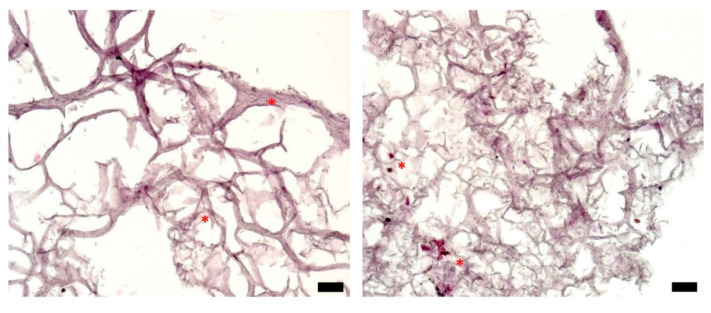
Histological analysis of representative sections of a triple-layered tissue construct (HE staining), day 0; optical microscopy, ob. 10×, scale bars = 10 µm. Stars highlight cells located in the bulk of the hydrogel.

Figure 4 presents typical sections of triple-layered constructs, prepared on day 0—the day of bioprinting. These were obtained by embedding the tissue constructs in a cryogenic medium, slicing them, and applying the HE staining protocol [32]. There are only a few cells on each section at the beginning of the experiment, distributed within the bioprinting gel matrix (stars). This is expected because of the relatively low cell density (10^6^ cells/mL) in the hydrogel delivered by the bioprinter (see Supplementary Material, Figure S4).

**Figure 5:**
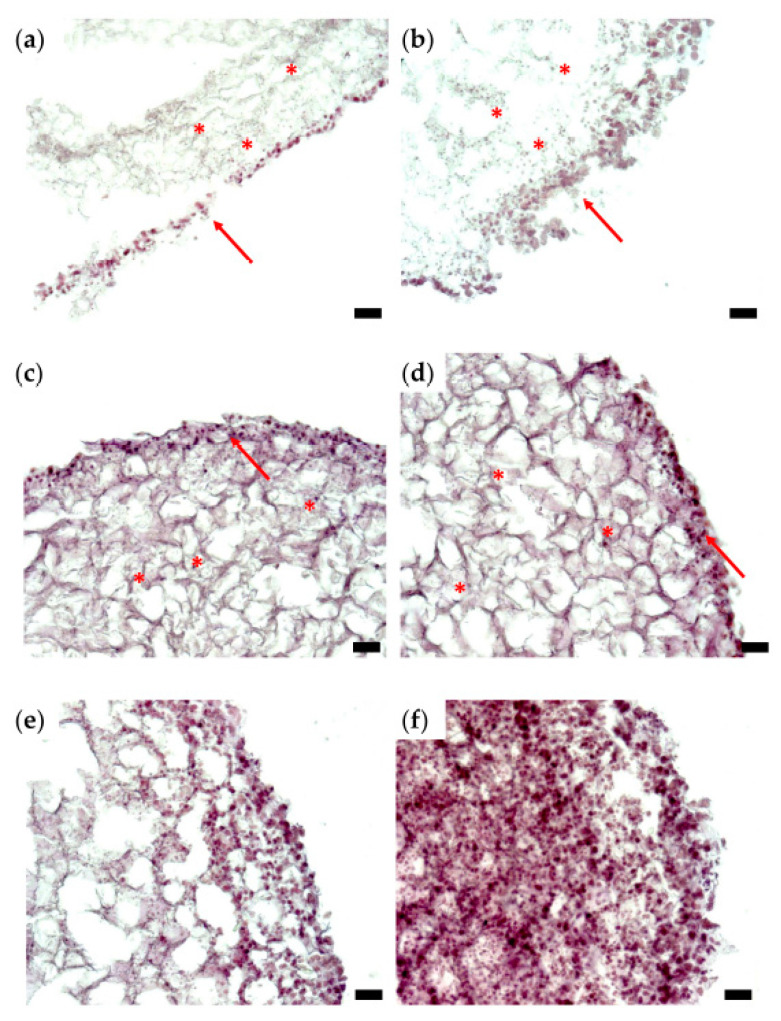
Histological analysis of representative sections of a triple-layered tissue construct (HE staining): (**a**,**b**) day 6; (**c**,**d**) day 10; (**e**,**f**) day 14; optical microscopy, ob. 10×, scale bars = 10 µm. Stars indicate cellular distribution on the inner part; arrows point to the exterior part of the structure, which is intensely populated with cells.

Cells attached to each other, as well as to the filaments of the hydrogel, can be distinguished in Figure 5a,b, which depict typical histological sections of tissue constructs evaluated after 6 days of culture. The cells are distributed within the inner part of the tumor structure (stars) but also tend to form a stratified capsule towards the outer part of the bioprinted construct (arrows). This uneven cellular distribution can also be attributed to cell movement along nutrient and oxygen gradients. Nevertheless, compared to day 0, on day 6, the cell count is increased in the overall structure. On day 10 of culturing the tumor construct (Figure 5c,d), secretion of the extracellular matrix can be evaluated by the decreased size of the hydrogel filaments network, as well as by the presence of numerous cells within the secreted matrix (stars). The nonuniform cellular distribution, with higher cell density towards the outer part of the structure, is maintained (arrows), as also observed on day 6 (Figure 5a,b). In the histological sections shown in Figure 5e,f, a triple-layered construct was evaluated after 14 days in culture. Here, cells are distributed within the entire construct, which is tending to form a fully populated tumor structure. There is a large discrepancy between the bioprinted tissues’ histological aspects on day 0 (Figure 4) and day 14 (Figure 5e,f), which indicates that the 3D cell culture conditions were favorable for cell growth and proliferation, thus forming a tumor tissue-like structure in vitro.

Although histological sections do not enable a quantitative assessment of the proliferation rate of cells in a 3D system, Figure 5 demonstrates that cells enclosed in the bioprinted constructs proliferate, move and remodel the hydrogel.

### 3.2. MMC Simulations of Multicellular Self-Assembly within the TME Constructs

**Figure 6:**
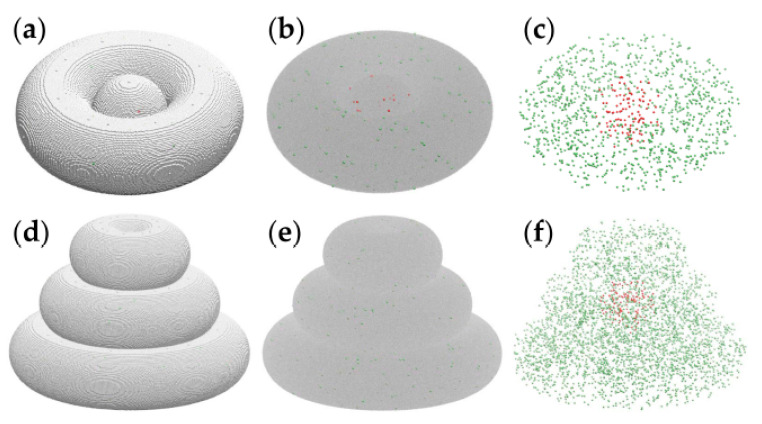
Computational models of the toroidal structure (**a**–**c**) and triple-layered structure (**d**–**f**), visualized using VMD [40]. Cancer cells are pictured by red spheres, whereas peritumoral cells are depicted as green spheres. (**a**) The toroidal tissue construct is represented on a cubic lattice, with 1.2 × 10^6^ cell-sized volume elements of the hydrogel pictured as silver spheres. (**b**) The toroidal structure with the hydrogel is shown as a translucent gray medium. (**c**) The cellular components of the toroidal tissue construct (i.e., hydrogel not shown): 109 tumor cells and 1033 peritumoral cells. (**d**) The triple-layered tissue construct, with about 3.8 × 10^6^ volume elements of hydrogel shown explicitly as silver spheres. (**e**) The triple-layered structure with the hydrogel is represented as a translucent continuum. (**f**) The 125 tumor cells and 3716 peritumoral cells are comprised by the computational model of the triple-layered structure.

Figure 6 depicts computational models of realistic size of the toroidal tissue construct (panels a–c) and the triple-layered tissue construct (panels d–f).

The model systems shown in Figure 6 represent the biological constructs at ascale of about 1:1. Indeed, the spheroid diameter and the pipe radius of the torus are equal to 60 cell diameters; assuming a cell diameter of about 10 µm, the model represents bioprinted constructs made of spheroids of 0.6 mm in diameter embraced by hydrogel threads of the same diameter.

The computational model describes the hydrogel as a set of interacting volume elements as large as cells are; these are depicted as silver spheres in Figure 6a,d. To account for the cell density prepared in experiments of 10^6^ cells/mL, we set the cell volume fraction to 10^−3^ and relied on a random number generator to distribute cells randomly within the hydrogel (see Figure 6, red and green spheres). Indeed, 1 mL = 1 cm^3^ = (10^4^ µm)^3^ = 10^9^ cell volumes, so if one out of 1000 particles represents a cell, then every mL of the cell–hydrogel mixture (10^9^ particles) contains 10^6^ model cells.

Cross-sections of the computational model contain just a few cells (Figure S4), in accord with the histological section of day 0 (Figure 4).

**Figure 7:**
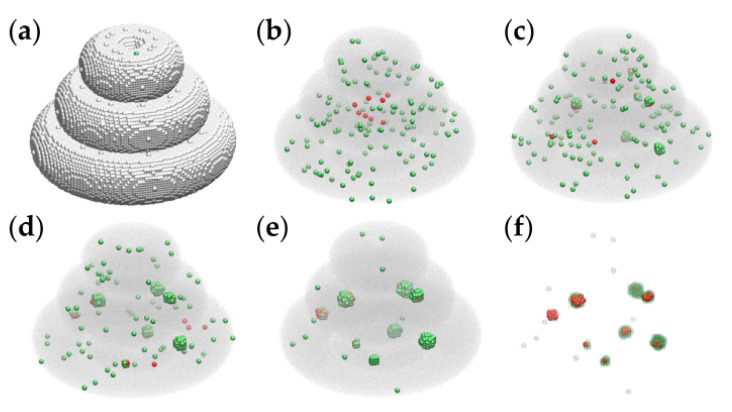
Representative MMC simulation snapshots of the evolution of a triple-layered tissue construct built on a cubic lattice at the scale of 1:3. Tumor cells and peritumoral cells are represented by red and green spheres, respectively. (**a**) The initial state containing about 1.4 × 10^5^ cell-sized volume elements of the embedding hydrogel is pictured as silver spheres. (**b**) The initial state, showing the hydrogel as a translucent gray medium, contains 8 tumor cells and 137 peritumoral cells; (**c**) the configuration obtained by running the simulation for 2 × 10^3^ MCS; (**d**) the result of 5 × 10^3^ MCS; (**e**) the outcome of 10^4^ MCS; (**f**) the result of 10^4^ MCS with the embedding hydrogel removed, and the peritumoral cells represented as transparent green spheres—to visualize the relative positions of malignant cells and peritumoral cells.

Computer simulations were conducted to investigate the hierarchy of interaction energies consistent with the spontaneous structure formation observed in bioprinted TME constructs. Given the large number (millions) of hydrogel volume elements present in the computational models of Figure 6, we simulated tissue construct evolution in smaller models, whose linear size is 3 times smaller than that of the realistic model. The initial state of these simulations is represented in Figure 7a,b.

We ran the simulations on desktop computers with the following specifications: Intel i5 CPU @ 3.40 GHz, 16 GB RAM, 64 bit, operating system (Windows 10 Enterprise). In the case of the triple-layered structure from Figure 7a**,** the running time was about 30 min for 10^4^ MCS.

The model parameters employed in the computer simulation of Figure 7 are given in the first row of Table 1 (set 1).

**Figure 8:**
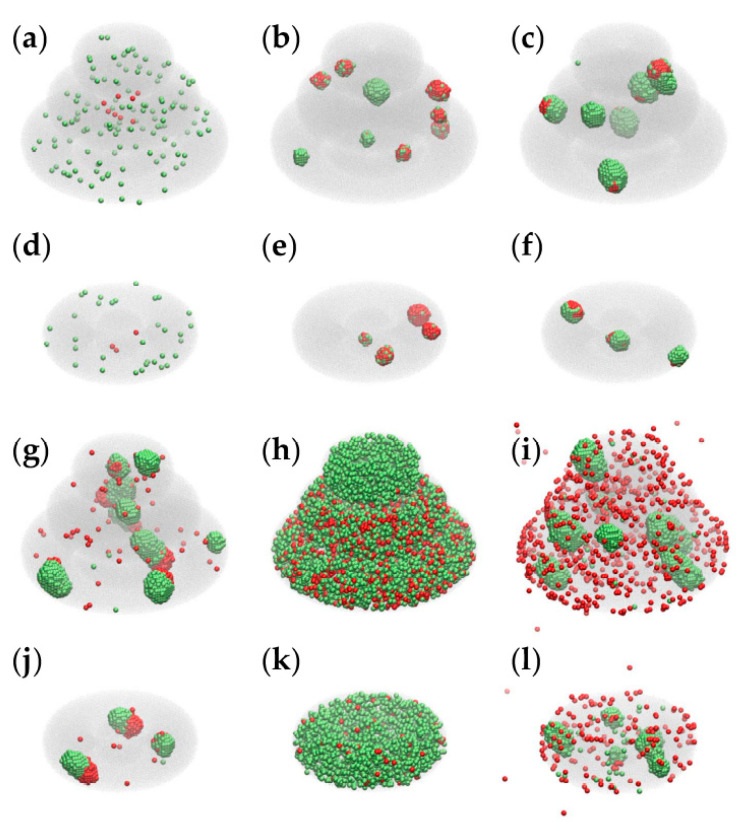
Results of MMC simulations performed with different model parameters. We used VMD [40] to depict the hydrogel as a translucent gray medium, peritumoral cells as green spheres and cancer cells as red spheres. (**a**) The initial state of the triple-layered structure; (**b**,**c**,**g**–**i**) the results of 2 × 10^4^ MCS performed for the triple-layered structure with parameter sets 1–5, respectively (Table 1); (**d**) the initial state of the toroidal structure; (**e**,**f**,**j**–**l**) the result of 2 × 10^4^ MCS conducted for the toroidal structure with parameter sets 1–5, respectively.

Figure 8 presents the impact of model parameters on the outcome of the MMC simulations for both models: triple-layered (a–c, g–i) and toroidal (d–f, j–l); the first two panels of the above lists Figure 8a,d depict the initial configurations of the two models, whereas the other panels are snapshots of the simulation taken after running 2×10^4^ MCS with the parameter sets 1 to 5, respectively (given in Table 1).

Parameter sets 1 and 2, listed in Table 1, lead to configurations that recapitulate certain features of the TME (in Figure 8b,e correspond to set 1, whereas panels **c** and **f** correspond to set 2). Both sets lead to aggregation and colocalization of cancer cells and peritumoral cells; set 1 ensures the infiltration of peritumoral cells in the tumor spheroid (a characteristic of immune cells [29]), whereas set 2 causes peritumoral cells to spread on the surface of cancer cell spheroids (as TAFs do [2]). Sets 3–5 lead to configurations that have no experimental counterparts: set 3 describes heterotypic aggregation, but none of the two cell populations tends to wrap the other one; set 4 does not lead to aggregation because of vanishing interfacial tensions between the cells and the hydrogel; set 5 drives the aggregation of peritumoral cells, whereas cancer cells are free to move within the hydrogel and also in the surrounding cell culture medium (in disagreement with experimental results on anchorage-dependent cells [2]).

Although the parameters that account for cell proliferation ($p_{2}$ and $p_{3}$) are the same in all 5 sets (Table 1), the emergent configurations differ in cell numbers because the computational algorithm only allows those model cells to proliferate that are next to the hydrogel or to the cell culture medium. The results of 5 × 10^4^ MCS are shown in Figure S5.

## 4. Discussion

This work presents 3D printed models of the TME consisting of cancer cells incorporated in a central spheroid wrapped in hydrogel rings loaded with TAFs and PBMCs. Unlike in the toroidal tissue construct, in the triple-layered structure, the cancer cells were surrounded on all sides by peritumoral cells uniformly distributed in the CELLINK hydrogel, giving rise to a more consistent microarchitecture of cell clusters separated by cell-free domains.

The hydrogel used in this work was not prepared in-house. Instead, we used a commercial one—the CELLINK universal bioink (CELLINK, Sweden). According to the manufacturer, this hydrogel contains a mixture of nanofibrillar cellulose, sodium alginate, D-mannitol and HEPES buffer. We considered this bioink for our 3D cell culture because several reports demonstrated that nanocellulose-alginate hydrogels mimic well the extracellular matrix properties while providing low cytotoxicity [41].

Histological data suggest that the cells present in the TME tissue constructs can remodel the hydrogel’s filament network via traction forces and the action of proteolytic enzymes—matrix-metalloproteinases (MMPs). This observation is consistent with findings related to cervical tumor models built by 3D bioprinting [20]. In their work, Zhao et al. used a hydrogel made of 10% gelatin, 1% sodium alginate and 2% fibrinogen to incorporate HeLa cells (10^6^ cells/mL). Cell spheroid formation was observed, 65% of them being larger than 50 µm in diameter after 5 days of culture. Moreover, cells cultured in 3D hydrogels secreted more than twice as much MMP-2 and MMP-9 asthose cultured in Petri dishes. Our work is similar to ref. [20] in what concerns the initial cell density of the bioprinted constructs and the presence of sodium alginate in the hydrogel. Despite differences in construct geometry, biomaterials, and cell types, the TME model presented in this work recapitulates several aspects of the structure formation observed by Zhao et al., suggesting that the 3D environment plays a central role in the self-organization of cancer cells. Nevertheless, the differences observed between the toroidal and triple-layered tissue constructs in the present work suggest that the topology of the TME is also important. The proximity of TAFs and PBMCs has facilitated cell proliferation in the triple-layered construct, resulting in higher cell density and more consistent structure formation.

The geometry of our toroidal tissue construct is similar to the glioblastoma-on-a-chip model developed by Yi et al. [18]. They employed cancer cells and endothelial cells suspended in hydrogel (5 × 10^6^ cells/mL) to print a flat, compartmentalized tumor-stroma model: a cancer cell-laden hydrogel disk surrounded by a concentric hydrogel ring comprising human umbilical vein endothelial cells (HUVECs). Importantly, the model tissue was enclosed in a microfluidic device that prevented gas and nutrient exchange from the top and bottom, thereby creating radial concentration gradients and central hypoxia. In our study, bioprinted constructs were bathed by cell culture medium in 24 well cell culture plates, so hypoxia could develop only in the triple-layered construct, in which tumor cells were buried in the bulk of the structure, at depths of the order of 0.8–1.5 mm. In their work [18], Yi et al. employed two types of hydrogel: collagen and another one prepared from decellularized pig brain extracellular matrix (BdECM). In BdECM, cancer cells had a higher proliferation rate and higher expression of pro-angiogenic factors and ECM-remodeling proteins. In light of these findings, we plan to investigate TME models made of cells suspended in different hydrogels.

To avoid the impact of the embedding hydrogel, Langer et al. investigated tumor heterogeneity using TME models built by scaffold-free bioprinting [15]. They printed a core of malignant cells wrapped in a shell of stromal cell mix (primary human mammary fibroblasts and HUVECs). A variety of TME models were obtained by using different breast cancer cell lines (MCF-7, SKBR3, HCC1143 and MDA-MB-231). Unlike in the present work, the bioink used in. [15] included tunable hydrogels to assure mechanical support during tissue construct fabrication, but these were removed during subsequent culture, leaving behind a pure cellular structure. The bioprinted constructs of about 2mm × 2mm × 1mm in size evolved spontaneously into a cancer cell spheroid covered by a shell of stromal cells. MMC simulations of multicellular spheroids composed of two cell populations of different cohesivities show, in accord with experiments [31], that the system spontaneously forms core–shell structure, with the more cohesive population being wrapped by, the less cohesive one [38]. A core–shell arrangement was also observed in 2D simulations of a high cell–density bioprinted model of the tumor microenvironment [42]. In the present study, post-printing self-assembly did not result in a concentric arrangement of the constituent cells, neither in experiments nor in simulations, presumably because of the much smaller cell density of the bioprinted TME constructs.

It is unclear why the cells were less organized in toroidal tissue constructs than in triple-layered ones. The experiments indicated a discrepancy between the post-printing evolution of the two constructs (Figure 3c,f), but the computational modeling did not reproduce it (Figure 8b,e). A distinctive feature of the triple-layered structure is that it gives rise to gas and nutrient gradients, akin to the in vivo TME, but these are not considered in the computational model. Further research will be needed to test the impact of oxygen and glucose gradients on the evolution of malignant and peritumoral cell populations. One interesting option in this respect is to include the toroidal structure into a tissue-on-chip device [18].

The TME plays a critical role in metastasis, too. In a recent study [43], Kok et al. demonstrated that fibrotic niche generation by metastatic cells is decisive for polyclonal metastasis. When nonmetastatic and metastatic cells were co-transplanted to the spleen, both cell types migrated into the liver within three days and formed colonies there. The metastatic cells activated hepatic stellate cells, inducing fibrotic niche generation. In this environment, nonmetastatic cells continued to proliferate even after the selective depletion of the metastatic cells. Kok et al. suggest that targeting the niche may be an effective strategy for preventing the spread of solid tumors [43]. Such hypotheses may be tested using model tissues that mimic the TME.

In the computational model employed in this work, the algorithm only enables superficial cells to proliferate (i.e., those cells located in the vicinity of hydrogel or cell culture medium). This requirement is a rough approximation of the experimental fact that cell proliferation mainly occurs close to the tumor periphery due to limited oxygen and nutrient availability within the bulk of the tumor [18,42].

The limitations of this study include (i) the relatively low cell density of the bioprinted construct, (ii) the lack of vascular cells in the TME, and (iii) the exclusive focus on cell adhesion in the computational analysis of cellular rearrangements. Extrusion bioprinting of bioinks with high cell density is feasible [15], although it would require far more cells. We plan to increase cell density by an order of magnitude in future studies. The incorporation of vascular cells is an appealing objective, too. Recent progress in 3D bioprinting [44,45,46] makes it possible to fabricate bulky tissue constructs with branched tubular structures within them; moreover, endothelial cells incorporated in the peritumoral environment self-organize into a microvasculature [18]. Hence, the TME models could be endowed with a complex vasculature. Cell source options for this task include HUVECs [11] and human-induced pluripotent stem cell (iPSC)-derived endothelial progenitors [47]. In this respect, immunological aspects also need to be considered. The computational analysis should be extended to describe more types of peritumoral cells, as well as oxygen and nutrient gradients. A 3D version of the approach of Bustamante et al. [42] seems to be a good starting point, although the differences between the algorithms employed in the cell-switching MMC model and the cellular Potts model make the implementation far from straightforward. If technical difficulties can be surmounted, the extended model might be able to predict the formation of a necrotic core in the center of large cell clusters, and cell proliferation might be simulated as a function of oxygen and glucose concentration.

## 5. Conclusions

This work presents tissue constructs fabricated by extrusion bioprinting as models of the tumor microenvironment (TME). The models proposed here differ from previous ones in geometry, matrix composition, and cellular composition. Our results confirm the hypothesis that scaffold-based models of the TME can be fabricated by extrusion bioprinting. Despite limited printer resolution, biomimetic structures emerge as a result of multicellular self-assembly.

The artificial hydrogel used in this study was appropriate for sustaining cell growth and proliferation. Cells migrated within the hydrogel and self-organized into heterotypic aggregates, mostly located on the periphery of the construct. Computer simulations suggest that the formation of aggregates comprising malignant cells and peritumoral cells can be explained by the differential adhesion hypothesis (i.e., by the tendency of the cells to form the largest possible number of strong bonds with one another as well as with the surrounding medium [31]). The superficial localization of the aggregates, however, was not captured by the simulations. Further investigations will be needed to establish whether it can be explained by taking into account oxygen and nutrient gradients within the bioprinted construct.

In vitro engineered TME models of diverse compositions are promising tools for fundamental research that aims to decompose the key players and mechanisms involved in tumor growth and migration. In addition, TME models built from patient-derived cells may be used to test the effectiveness of anti-cancer therapies, thereby devising personalized treatment plans.

## Figures and Tables

**Table 1:** Computer simulation parameters.

Set	Works of Cohesion and Adhesion ^1^	Interfacial Tensions ^2^	$p_{2}$	$p_{3}$
1	0.0, 0.8, 2.4, 3.2, 0.0, 0.0, 0.0, 0.9, 0.8, 3.1	0.4, 1.2, 1.6, 0.7, 1.2, −0.3	2 × 10^−4^	2.4 × 10^−4^
2	0.0, 0.8, 2.4, 3.2, 0.0, 0.0, 0.0, 0.9, 0.8, 2.1	0.4, 1.2, 1.6, 0.7, 1.2, 0.7	2 × 10^−4^	2.4 × 10^−4^
3	0.0, 0.6, 2.8, 2.8, 0.0, 0.0, 0.0, 1.2, 1.2, 2.2	0.3, 1.4, 1.4, 0.5, 0.5, 0.6	2 × 10^−4^	2.4 × 10^−4^
4	0.0, 0.8, 2.4, 3.2, 0.0, 0.0, 0.0, 1.6, 2.0, 2.1	0.4, 1.2, 1.6, 0.0, 0.0, 0.7	2 × 10^−4^	2.4 × 10^−4^
5	0.0, 1.4, 2.2, 0.8, 0.0, 0.0, 0.0, 1.4, 0.7, 0.2	0.7, 1.1, 0.4, 0.4, 0.4, 1.3	2 × 10^−4^	2.4 × 10^−4^

^1^ Listed in the following order: $\varepsilon_{00},\varepsilon_{11},\varepsilon_{22},\varepsilon_{33},\varepsilon_{01},\varepsilon_{02},\varepsilon_{03},\varepsilon_{12},\varepsilon_{13},\varepsilon_{23}$. ^2^ Listed as: $\gamma_{01},\gamma_{02},\gamma_{03},\gamma_{12},\gamma_{13},\gamma_{23}$.

## Data Availability

The data generated during this study are available from the corresponding author upon reasonable request.

## Supplementary Materials

The following are available online at https://www.mdpi.com/article/10.3390/mi12050535/s1, Figure S1: Scheme of the study design, Figure S2: Digital models of tissue constructs that mimic the tumor microenvironment, Figure S3: Picture of the actual bioprinting process; Figure S4: Computational models of the tissue constructs shown in cross-section to illustrate the relatively low number of cells in each section, Figure S5: Snapshots of representative computer simulations of the evolution of bioprinted model tissues shown in axial cross-section, Video S1: The bioprinting process illustrated by 3D printing of ultrasound transmission gel onto the lid of a 24-well cell culture plate.
