# Supplementary material for: 3D Bioprinting of Model Tissues That Mimic the Tumor Microenvironment
Source: Micromachines (Basel). 2021 May 9;12(5):535. doi: 10.3390/mi12050535 (PMC8151644; doi:10.3390/mi12050535)
Supplement: Supplementary file 1 [file micromachines-12-00535-s001.zip › Supplementary Materials_proofreading.pdf]

Supplementary Materials:

3D Bioprinting of Model Tissues that Mimic the Tumor Microenvironment

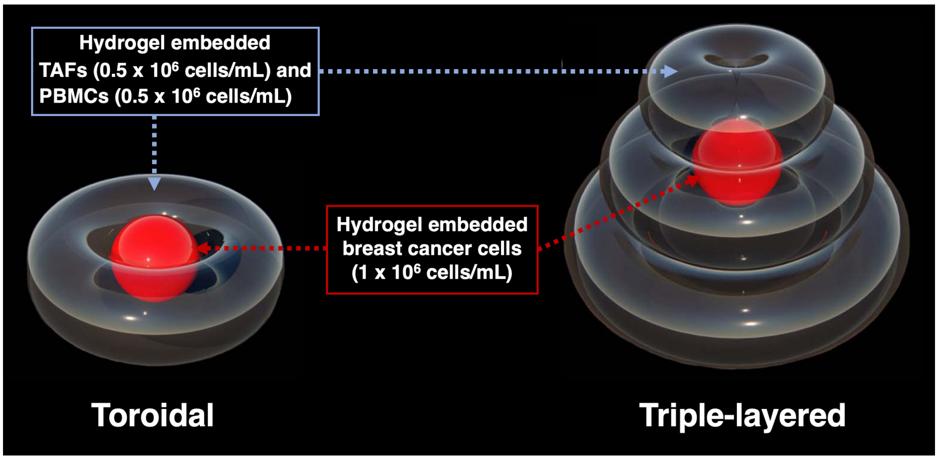

Figure 1. Scheme of the study design.

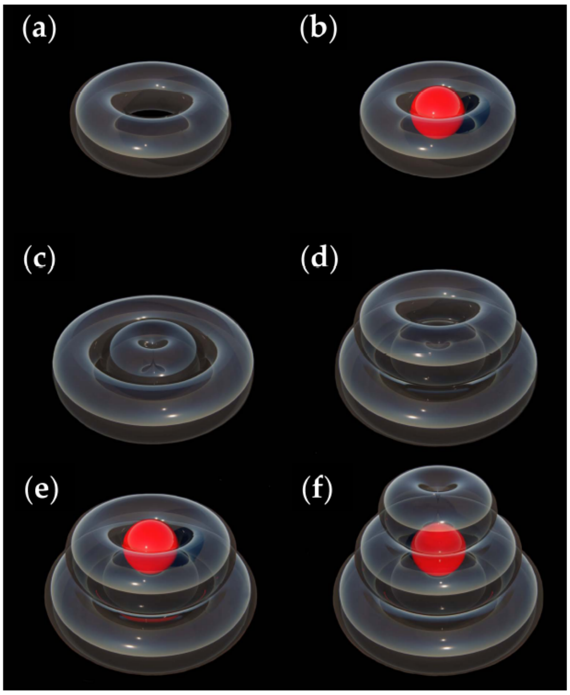

Figure S2. Digital models of tissue constructs that mimic the tumor microenvironment: the toroidal structure (b) and the triple-layered structure (f); here, transparent tori represent rings of hydrogel loaded with peritumoral cells, whereas the red sphere stands for a cancer-cell-laden hydrogel droplet. (**a**) The model of the structure delivered by the first print head; (**b**) The model of the toroidal structure completed by the second print head; (**c**) The base layer; (**d**) The first item of the second layer; (**e**) The first two layers completed; (**f**) The triple-layered structure.

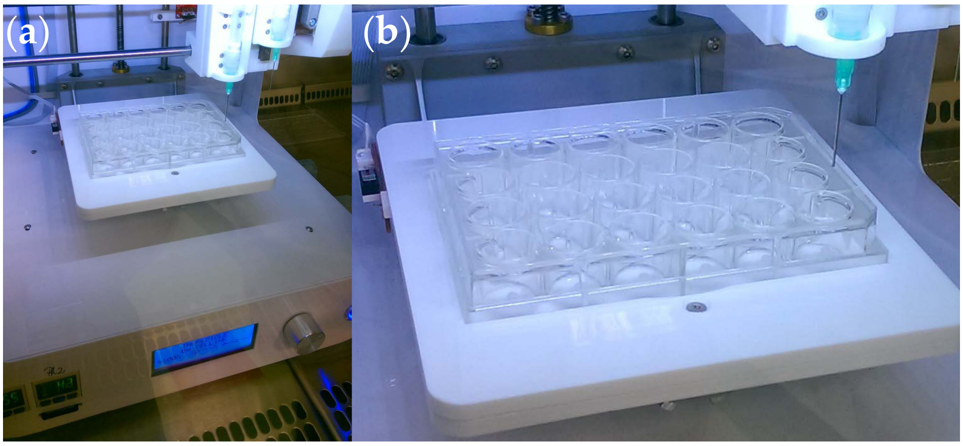

Figure S3. Picture of the actual bioprinting process. (**a**) Digital photograph of the INKREDIBLE bioprinter (CELLINK, Sweden) placed in a sterile cell culture hood, taken during the bioprinting of the hydrogel loaded with peritumoral cells; the digital displays from the lower-left corner show the pressures applied to the print heads (PH) – 55 kPa for PH_1 and 42 kPa for PH_2); (**b**) A zoom-in picture of the 24-well cell culture plate during the printing of the base layer (according to the model depicted in Figure S2d).

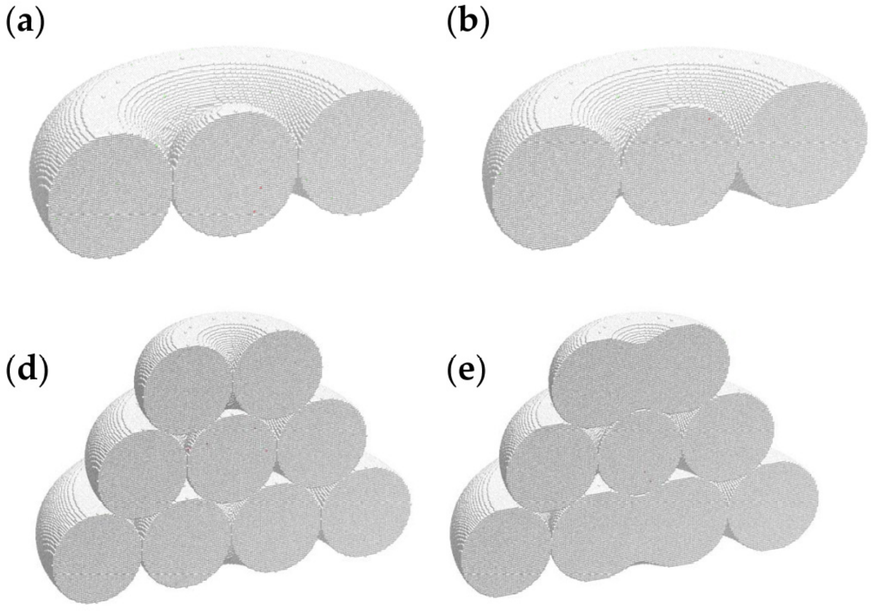

Figure S4. Computational models of the tissue constructs shown in cross-section to illustrate the relatively low number of cells in each section. Visualization was done in VMD [40]. Here, silver spheres depict hydrogel volume elements, whereas green and red spheres represent peritumoral cells and cancer cells, respectively. These models were built on a 3D cubic lattice and represent the corresponding biological systems at a scale of 1:1, at a cell density of 10^6 cells/mL, used in experiments (see main text for details). (**a**) Axial cross-section of the toroidal structure; (**b**) A representative vertical cross-section of the toroidal structure; (**c**) Axial cross-section of the triple-layered structure; (**d**) A typical vertical cross-section of the triple-layered structure.

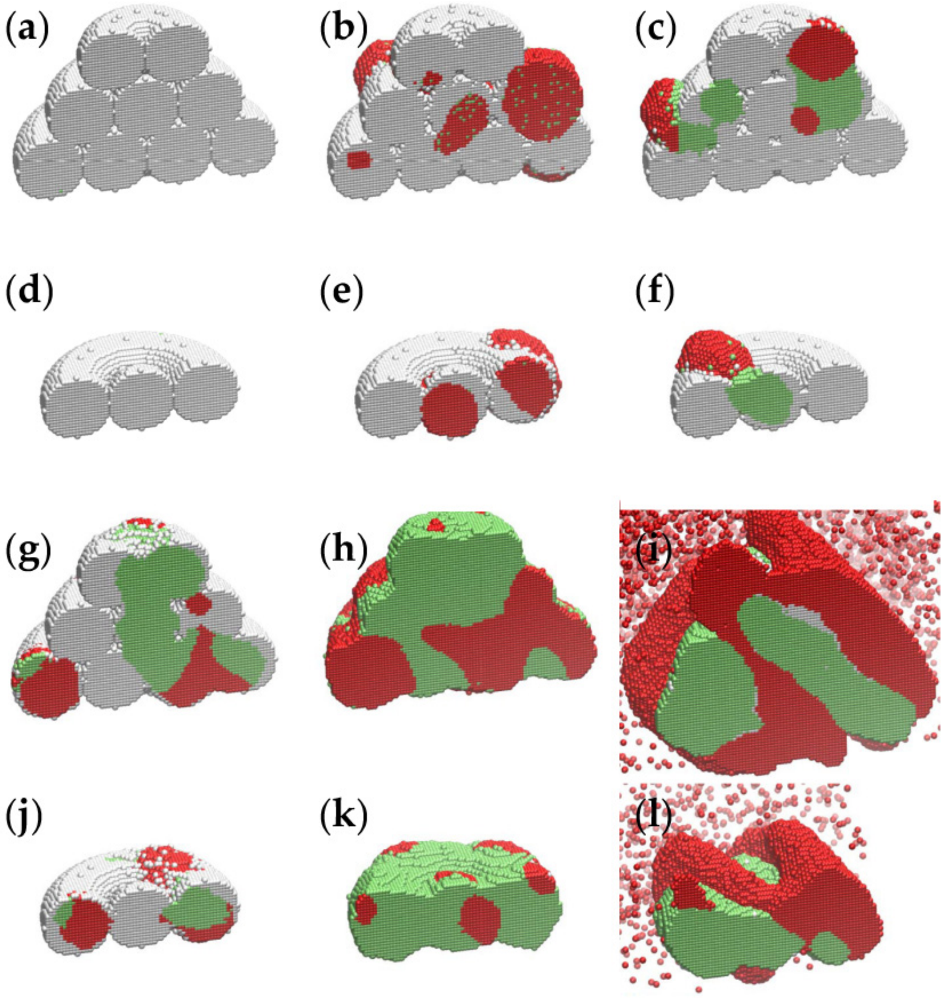

Figure S5. Snapshots of representative computer simulations of the evolution of bioprinted model tissues shown in axial cross-section. (**a**) The computational model of the initial state of the triple-layered structure (scale 1:3); (**b**, **c**, **g**, **h**, **i**) The outcome of 5×10^4 MCS performed on the model of the triple-layered structure with parameter sets 1-5, respectively (listed in Table 1); (**d**) The initial state of the toroidal structure (scale 1:3); (**e**, **f**, **j**, **k**, **l**) The result of 5×10^4 MCS conducted on the model of the toroidal structure with parameter sets 1–5, respectively. Visualization is described in the caption of Figure S2.

Video S1: The bioprinting process illustrated by printing ultrasound transmission gel onto the lid of a 24-well cell culture plate. Here, the transparent gel – Konix ultrasound transmission gel (Turkuaz, Turkey) – represents the peritumoral medium, whereas the same gel stained with Neutral Red dye (Sigma-Aldrich, Germany) represents the droplet of hydrogel loaded with cancer cells.
